# The correlation between two occlusal analyzers for the measurement of bite force

**DOI:** 10.1186/s12903-022-02484-9

**Published:** 2022-11-05

**Authors:** Yi-Fang Huang, Chin-Man Wang, Wann-Yun Shieh, Yu-Fang Liao, Hsiang-Hsi Hong, Chung-Ta Chang

**Affiliations:** 1 Department of General Dentistry, Chang Gung Memorial Hospital, 33305 Linkou, Taiwan; 2 School of Dentistry, College of Oral Medicine, Taipei Medical University, 11031 Taipei, Taiwan; 3 Graduate Institute of Dental and Craniofacial Science, College of Medicine, Chang Gung University, 33302 Taoyuan, Taiwan; 4 Department of Physical Medicine and Rehabilitation, College of Medicine, Linkou Chang Gung Memorial Hospital, Chang Gung University, 33305 Taoyuan, Taiwan; 5 Department of Computer Science and Information Engineering, College of Engineering, Chang Gung University, 33302 Taoyuan, Taiwan; 6 Department of Physical Medicine and Rehabilitation, Chang Gung Memorial Hospital, 33302 Taoyuan, Taiwan; 7 Department of Craniofacial Orthodontics, Chang Gung Memorial Hospital, 10507 Taipei, Taiwan; 8 Craniofacial Center, Chang Gung Memorial Hospital, 33378 Taoyuan, Taiwan; 9 Craniofacial Research Center, Chang Gung Memorial Hospital, 33305 Linkou, Taiwan; 10 College of Medicine, Chang Gung University, 33302 Taoyuan, Taiwan; 11 Department of Periodontology, Chang Gung Memorial Hospital, 33305 Linkou, Taiwan; 12 Department of Emergency Medicine, Far Eastern Memorial Hospital, No. 21, Sec. 2, Nanya S. Rd., Banciao Dist, 22056 New Taipei, Taiwan; 13 Graduate Institute of Medicine, Yuan Ze University, 32003 Taoyuan, Taiwan

**Keywords:** Occlusal indicator, Occlusal force, Bite, Quantify, Pressure sensitive film sheet

## Abstract

**Background:**

Occlusal force represents masticatory function. Using quantifiable occlusal indicators provides a more objective occlusal force evaluation. In the recent dental practice, digital methods such as the Dental Prescale II (DP2, GC Corp., Tokyo, Japan) and T-scan (T-Scan III v8; Tekscan Inc.) are commonly used in clinics to evaluate treatment outcomes. The T-scan provides the relative bite force (%) compared to the maximal bite force on individual teeth or the unilateral arch. The DP2 can quantify occlusal force, measured in newtons (N), on the half arch or the overall bite, but it is difficult to identify the bite force on an individual tooth. It is difficult to select a device that fulfils all the requirements to record occlusal force. This study aimed to investigate the association between the bite measured by the DPS2 and T-scan to determine whether the measured bite force is comparable through calculation.

**Methods:**

A total of 80 healthy adults, including 41 women and 39 men with a mean age of 38.2, were requested to bite pressure sensitive film sheets ten minutes apart. Linear regression analysis was used to estimate the measured bite force by the DP2 and T-scan.

**Results:**

There was a significant positive correlation between the occlusal force measured by the DP2 and T-scan (*P* < 0.01) when intercept was equal to zero as confounders were adjused. These results provided the comparability of the measured occlusal forces determined by the DP2 and T-scan.

**Conclusion:**

The estimated bite force determined by DP2 and T-Scan is convertible using the linear equation from this study to increase the value for clinical applications. The estimated bite force from the two quantifiable occlusal indicators are comparable. The two commercially available quantifiable occlusal indicators can be fully adapted to all clinical requirements according to this result.

## Background

Occlusal force represents the function and efficacy of mastication; it has been recorded as a variable to evaluate the outcome of various dental treatments [1]. According to the glossary of prosthodontics terms [2], dental occlusion is defined as, “the static relationship between the incising or occlusal surfaces of the maxillary or mandibular teeth or tooth analogues.” With occlusal stability, multiple even contacts are created between the teeth and antagonist [3]. The occlusion should be as balanced and as stress free as possible. For years, occlusal contacts have been registered to determine their exact location by various materials and methods. The methods used to record occlusal-articulation relations are qualitative and quantitative [4]. Articulating papers are a common method to determine occlusion in clinical practice, and are used to perform occlusal adjustments during prosthodontic treatments. However, articulating paper can only provide the occlude contact, but not quantify the bite force or reveal the sequence of teeth contact [5]. Previous study indicated that there is no correlation between the size of the mark areas and the applied occlusal load [6]. Its reliability is often disputed because this indicator often produce false positive results or fail to record any occlusal contacts [7]. The limitation of this conventional occlusal record is the high degree of subjectivity during implementation. To measure the occlusal force more scientifically, quantifiable occlusal indicators have been developed.

This conventional qualitative method contains no scientific association between the depth of the color and the mark, the amount of force, the surface area, or the simultaneous contact sequence when the results are represented as a paper mark [8]. The validation for developing a quantitative occlusal indicator requires improvement of the current inadequacies of conventional qualitative registration approaches. In the recent dental practice, digital methods can give more accurate and reliable data on the registration of occlusal contacts [3]. The quantifiable occlusal indicators used to record bite force have a variety of designs and working principles. The different analyzing systems can be used to objectively measure relative or real quantitative occlusal forces, as well as the timing or sequence of tooth contact [9]. The Dental Prescale II (DP2, GC Corp., Tokyo, Japan), introduced in 2018, is often used to measure the bite force of the entire dentition in clinical practices [10]. Previous investigations proposed various viewpoints related to the maximum occlusal force by the DP2 that has been widely used as an important parameter that can objectively evaluate the masticatory function [11]. Multiple investigations performed with the DP2 validated it as a reliable and reproducible system [10, 12]. However, that does not offer any information about the timing of the occlusal contact sequence, or the temporal sequence of the bite force buildup. The T-scan (T-Scan III v8; Tekscan Inc.) is an accurate and reliable digital dynamic occlusal analysis to objectively estimate occlusal contact [13]. The T-scan can only reveal the relative bite force compared to individual maximal occlusal force, which is represented as a percentage (%). The occlusal contact sequence from the first tooth contact to maximum intercuspation is recorded, and then played in real-time on the computer screen with the intensity of the associated force [13]. In a previous study, the T-scan could only be used to compare the change of occlusion in the same subject because it provided the relative occlusal force [5]. The DP2 reveals the quantified bite force represented in newtons which compares the difference of occlusal forces between subjects, but it cannot reveal the sequence and timing of tooth contact. It is difficult to select a device which fulfils all the requirements to record occlusal force. Hence, it is important to integrate the occlusal analysis from different devices to gain more information. If the measured bite force of different quantifiable occlusal indicators can be determined through calculation, the value for clinical applications can be greatly improved. The study aimed to investigate the correlation between the outcomes derived from the DP2 and T-scan.

## Methods

### Patient selection

The participants all had good communication skills and cooperated well in this study. All subjects were restricted to having good oral functions and displaying healthy periodontal conditions without neuromuscular disorders. Their age range was limited between 20 and 65 years-old. Subjects who had a history of temporomandibular disorders, head and neck cancer, stroke, or radiotherapy history were all excluded from this study. This study was approved by the Ethical Committee of Chang Gun Memorial Hospital (201204900B0). The subjects were only enrolled in this study after they had understood the aim of this investigation and signed the informed consents. We have confirmed all methods were carried out in accordance with relevant guideline and regulations in the “Ethics approval and consent to participate” section under “Declarations”.

### Bite force evaluation with Dental Prescale II and T-Scan III

The DP2 consists of a 150 μm thickness pressure-sensitive film sheets comprising three layers of polyethylene terephthalate (PET) covering a developer layer and a microcapsule layer. The microcapsules vary in size and thickness and are filled with color-producing ingredients that release a dye in response to the applied pressure ranging from 10 MPa to 120 MPa [10]. When the film sheet takes on occlusal loading force as teeth compress its matrix, the dye reacts with the developer layer to turn a red color, where the shade of the color is in proportion to the number of microcapsules breaking under the applied pressure. To scan the film after recording, a color image scanner analyzer (Bite Force Analyzer; GC, Tokyo, Japan) is used to estimate the bite force (N), the occlusal contact area (mm^2^), and the bite pressure (MPa) [12].

**Fig. 1:**
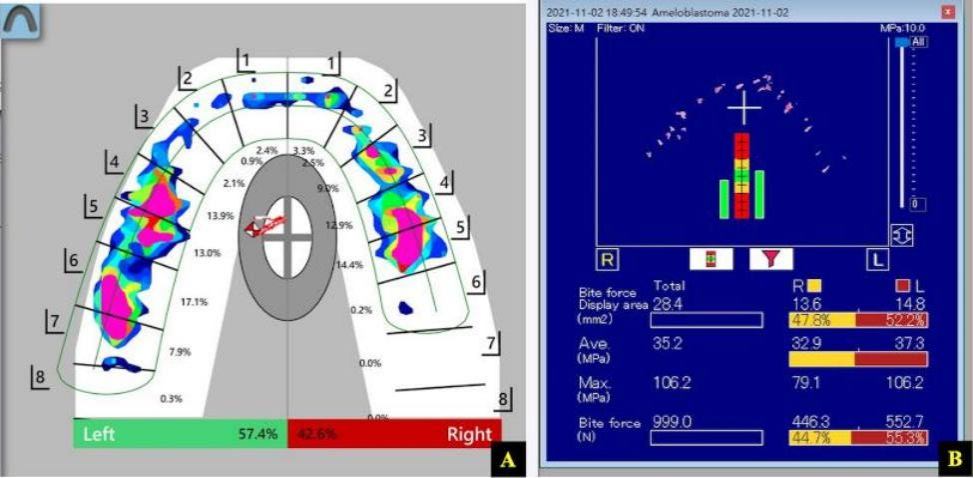
The bite force measured by different quantifiable occlusal indicators. The recorded comparison of the occlusal indicators on the left and right sides, including the percentage of bite force of the maximal occlusal force from the T-scan (**A**) and the quantitative bite force in newtons from the DPS2 (**B**).

The T-scan consists of a hand-held device with a 60 μm thick pressure measuring sensor that contains 1,500 compressible sensitive receptor points made of conductive ink. When the bite force is applied on the sensor, the electrical resistance of the conductive sensor is lessened because the force applied compresses the particles together to record the quantitative force data. Both the accurate and reliable computerized occlusal analysis systems T-scan III and DP2 were used to estimate the bite force. These two occlusal indicators consist of a tactile sensor, but the T-scan evaluates the relative level of bite force whereas the DPS2 measures the occlusal force in newtons. The subjects were asked to sit in a relaxed and upright position during occlusal parameter estimation. After the participants were familiar with the instructions, they were trained to firmly bite down on the sensor to reach maximal intercuspation for three seconds [14]. The subjects were requested bite the T-scan first then take a rest for then minutes then bite the DP2 so that each subject used both sensors during the same appointment. Figure 1 shows the recorded comparison of the occlusal indicators on the left and right sides, including the percentage of bite force of the maximal occlusal force from the T-scan and the quantitative bite force in newtons from the DPS2.

### Statistical analysis

To provide a desired significant level, effect size, and statistical power, a power analysis was used to estimate the minimum sample size required in this study. The data were represented as the mean and standard deviation (SD). A linear regression analysis was used to estimate the association between the relative bite force of the T-scan and the quantitative occlusal force revealed from the DPS2. SAS software version 9.4 (SAS Institute, Cary, NC) was used for these analyses, and a two-tailed test *P* < 0.05 was considered statistically significant.

## Results

A total of 80 participants including 41 women and 39 men were enrolled in this study. Their ages ranged from 25 to 45 years (average, 38.2 years). The distribution of the occlusal indicators on the right and left sides from the DP2 and the T-scan is revealed in Table 1. Based on the result of power analysis, to provide significant power the predictor variables were adjusted in the linear regression model which let intercept be equal to zero. Table 2 indicates the significant linearity between the occlusal force over the right and left arches measured by the DP2 and T-scan (the linear regression equation of right and left sides: y = 25.082x; y = 26.548x, *P* < 0.01).

**Table 1:** The distribution of relative and quantitative bite force on the right and left side measured by the different occlusal indicators

Occlusal indicators	Variable	Mean	SD	Median	Q1, Q3
DPS2	Right force (N)	1317.71	1093.44	989.7	747.2, 1458.58
	Left Force (N)	1376.2	899.85	1190.4	581.6, 2187.9
T-Scan	Right Force (%)	52.35	21.87	46.24	34.77, 63.60
	Left Force (%)	50.17	19.31	56.02	40, 65.48

Abbreviations: DPS2, Dental Prescale II; SD, Standard Deviation

## Discussion

The clinical concept of occlusion contains dynamic interactions of the mandibular movement and static morphological teeth contact interactions [15]. Even though there are many digital dynamic occlusal indicators to measure the bite force in the clinic, each quantitative occlusal analyzer has different limitations. The connection of the data as measured by different equipment to achieve maximal benefit is an important issue. This study preliminarily revealed the closely correlation between the DP2 and T-scan measurements. This study presented the close association between the occlusal analysis results from the DPS2 and the T-scan, and revealed the linear regression equation of these two occlusal indicators. The clinical significance of these results is that the data from these two occlusal analyzers can be used interchangeably to increase the value for clinical applications.

All disciplines of dentistry require that the Clinicians evaluate the articulation of the teeth with respect to simultaneous contacts, bite force, and timing. Conventional occlusal registration technology such as articulating paper lacks objective accuracy, reliability, and reproducibility [15]. Quantitative occlusal analysis techniques have been developed to overcome these limitations, and the DPS2 and T-Scan are the most common quantitative occlusal indicators used to estimate the occlusal relationship [16, 17]. The T-scan is often used to estimate the subject’s occlusal force because the T-scan system is not able to measure the absolute bite force [15]; it only presents the percentage of the bite force compared to the maximal occlusal force. Although the DP2 quantifies the bite force in newtons on the whole dentition or half arch, it is difficult to identify each individual tooth. This occlusal indicator also lacks the dynamic tooth contact sequence results so occlusal interference, such as premature contact, cannot be detected with this equipment. Both the DP2 and T-scan have their advantages and limitations to quantify occlusal force. The challenge many Clinicians face is the increased chair time to be familiar with the quantitative occlusal indicators. This study innovatively proposed a positive correlation between the measured occlusal forces determined by the DP2 and T-scan (Table 2). Through the linear regression equation derived from this study, the results data of the T-scan can be converted into the bite force represented as newtons. The relative bite force estimated from the T-scan could be used to compare the occlusal changes between the subjects. This investigation provided valuable clinical DP2 and T-scan applications. It is hoped that quantifiable occlusal force measurements become easier to apply and more popular in in the clinic.

**Table 2:** The association between DPS2 and T-Scan with linear regression analysis

Variable DPS	Variable T-Scan	Estimate	95% CI	***P***	Regression linear equation	Power Analysis	R^2^
Right force (N)	Right force (%)	25.082	17.195, 32.969	< 0.001^**^	y = 25.082x	0.99	0.7
Left force (N)	Left force (%)	26.548	19.163, 33.933	< 0.001^**^	y = 26.548x	0.99	0.76

Intercept = 0

Abbreviations: DPS2, Dental Prescale II; CI, Confident interval

*P < 0.05

This study pilot indicated the significant linearity between the quantifiable occlusal measurements of the DP2 and T-scan but there are multiple limitations. The T-scan can only reveal relative bite force compared to the maximal occlusal force on each tooth and half arch. It is difficult for the DP2 to identify each tooth contact representing an individual tooth. Hence, this study only could take the fact that the DP2 and T-scan both measure unilateral occlusal force in common so the results only could compare the quantified bite force of the right and left arch halves. Another limitation is the small sample size, which restricts the accuracy of the linear regression equation so these results can only compare the correlation of the quantified occlusal forces determined the DP2 and T-scan. To raise the realism of the linear regression equation between these two quantifiable occlusal indicators, further studies need to increase their sample size.

## Conclusions

Commercially available quantifiable occlusal indicators cannot be fully adapted to all clinical requirements. The T-scan provides the relative bite force percentage compared to maximal bite force on individual teeth or the unilateral arch. The DP2 quantifies the occlusal force in newtons on the half arch or the overall bite but it is difficult to identify the bite force on an individual tooth. There was a significant positive correlation between the occlusal force measured by the DP2 and T-scan. It is possible to compare the bite force estimated by the DP2 and T-scan by applying a correction using the regression equation obtained in this study. These results provided a correlation comparison of the measured occlusal forces determined by the DP2 and T-scan. The estimated bite force from the two quantifiable occlusal indicators are comparable.

## Data Availability

The data sets generated and/or analyzed during the present study are available from the corresponding author on reasonable request.

## Abbreviations

DP2: Dental Prescale II.
PET: polyethylene terephthalate.
SD: standard deviation.
